# Alanine-mediated P cycle boosting enhances the killing efficiency of kasugamycin on antibiotic-resistant *Xanthomonas oryzae*

**DOI:** 10.3389/fmicb.2023.1160702

**Published:** 2023-04-18

**Authors:** Yi Guan, Meiyun Lin, Peihua Shen, Zhenyu Zou

**Affiliations:** Fujian Key Laboratory of Marine Enzyme Engineering, College of Biological Science and Engineering, Fuzhou University, Fuzhou, China

**Keywords:** *Xanthomonas oryzae*, kasugamycin resistance, metabolomics, pyruvate cycle, exogenous alanine

## Abstract

The outbreak of Bacterial blight (BB) caused by *Xanthomonas oryzae* (Xoo) generates substantial economic losses to agricultural production. Antibiotics application is a valuable measure to control this bacterial disease. However, microbial antibiotic resistance dramatically reduced antibiotic effectiveness. Identifying the resistance mechanism of Xoo to antibiotics and restoring antibiotic susceptibility is one of the crucial ways to solve this problem. This study employed a GC-MS-based metabolomic approach to reveal the differential metabolomics between a kasugamycin-susceptible Xoo strain (Z173-S) and a kasugamycin-resistant strain (Z173-R_KA_). The metabolic mechanism of kasugamycin (KA) resistance in Xoo by GC–MS showed that the downregulation of the pyruvate cycle (P cycle) is a crucial feature of Z173-R_KA_ resistance to KA. This conclusion was confirmed by the decreased enzyme activities and the related gene transcriptional level in the P cycle. Furfural (an inhibitor of pyruvate dehydrogenase) can effectively inhibit the P cycle and increase the resistance of Z173-R_KA_ to KA. Moreover, exogenous alanine can reduce the resistance of Z173-R_KA_ to KA by promoting the P cycle. Our work seems to be the first exploration of the mechanism of KA resistance in Xoo by GC–MS-based metabonomics approach. These results provide a new idea for developing metabolic regulation to address KA resistance in Xoo.

## Introduction

Rice is one of the most widely grown food crops, feeding more than 50% of the world’s population. Its yield and quality are related to the survival and development of humankind (Ke et al., 2019). However, diseases caused by various bacteria, fungi and viral pathogens affect rice yield (Gnanamanickam et al., 1999). The rice blight (bacterial blight, BB) caused by *Xanthomonas oryzae* pv. *oryzae* (Xoo), a Gram-negative bacterial species in the *Gammaproteobacteria*, is one of the most destructive bacteriosis in rice farming, which may result in more than 50% yield decline (Mew, 1987; Liu et al., 2014; Chen et al., 2016; Li et al., 2018).

Currently, pharmaceutical agent usage is considered an efficient method for BB disease control. Antibiotic drugs are most widely applied, such as kasugamycin, zhongshengmycin, streptomycin, resveratrol, zinc thiazole and bismerthiazol (Ming et al., 1991; Jiang et al., 2003; Xu et al., 2013; Chen et al., 2015; Luo et al., 2020; Slack et al., 2021). Kasugamycin (KA herein), also called *haruamycin*, is an aminoglycoside antibiotic produced by *Streptomyces kasugaensis*, which was first isolated from Haruhi’s soil Shrine, Nara Prefecture, Japan, in April 1964 (Umezawa et al., 1965). KA binds to the 30S small subunit of the bacterial ribosome and acts on the A site of the 16S rRNA decoding region of the small subunit, thereby specifically inhibiting the binding of aminoacyl-tRNA to the mRNA-ribosomal protein complex, preventing the protein initiation of translation and inhibiting bacterial growth (Levitan, 1967; Kim et al., 2000; Berenjian et al., 2015). Compared with other antibiotics, KA has a broad bactericidal spectrum, strong internal absorption permeability, significant biological activity, and is relatively environmentally friendly (Ishiyama et al., 1965). Therefore, it is a pollution-free agricultural pesticide recommended by the Ministry of Agriculture and Rural Affairs of China and is widely used to control BB worldwide (Slack et al., 2021).

Although the widespread use of antibiotics can effectively prevent rice diseases, microbial antibiotic resistance dramatically reduces antibiotic effectiveness. Nowadays, the misuse and/or improper use of antibiotics has led to the emergence and rapid spread of antibiotic strains of Xoo (Ogawa, 1993; Xu et al., 2010; Li et al., 2018). Therefore, seeking adequate solutions to fight antibiotic resistance is urgent for bacterial disease control in Xoo. Developing new antibiotic agents is a feasible way. However, the development process is time-consuming, and the development pipeline is exhausted (Luepke and Mohr, 2017). Hence, exploring new solutions to control antibiotic-resistant Xoo is necessary to ensure the safety of rice production.

Metabolism is the primary feature of life that reflects the dynamic life function directly. Metabolomics, the systematic quantitative study of metabolites in organisms, enables us to instantly and accurately grasp the phenotype information of organisms (Nicholson and Lindon, 2008). Recently, metabolomics was applied in the microbial antibiotic-resistance research field. The inhibited TCA cycle and glyoxylate cycle have been proven to be the key mechanisms for the resistance of *Mycobacterium tuberculosis* to ciprofloxacin (Knoll et al., 2021); downregulation of amino acid biosynthesis and upregulation of cysteine and methionine metabolism are important mechanisms leading to the resistance of *Escherichia coli* to various antibiotics (Lin et al., 2019); enhancement of fatty acid biosynthesis pathway is associated with the acquisition of ciprofloxacin resistance in *Edwardsiella tarda* (Su et al., 2021). In addition, various exogenous substances were identified that can affect the metabolic pathways and change the metabolic phenotype to enhance or weaken some metabolic functions. For example, citrulline and glutamine promoted the TCA cycle to produce more NADH and reversed the resistance of *Salmonella* to apramycin (Yong et al., 2021). Nitrite promoted the nitrite-dependent NO biosynthesis and increased the susceptibility of clinically and laboratory-evolved *Pseudomonas aeruginosa* to cefoperazone-sulbactam sodium (Kuang et al., 2021b). Fructose activated the TCA cycle to generate more proton motive force to increase antibiotic absorption and restore sensitivity to kanamycin in multidrug-resistant bacteria (Su et al., 2015). Therefore, metabolomics analysis methods will provide new ideas for bacterial drug resistance control.

Kasugamycin is a widely applied antibiotic for controlling Xoo in the agricultural field. In the present study, we analyzed the metabolome of KA-resistant strain Z173-R_KA_ and susceptible strain Z173-S by the GC–MS-based metabolomic analysis. We demonstrated that the depression of the P cycle is a characteristic of the KA-resistant strain, and exogenous alanine can promote the P cycle and further enhance the sensitivity of Z173-R_KA_ to KA. To our knowledge, this is the first exploration to study the mechanism of KA resistance in *Xanthomonas oryzae* by metabonomics based on GC–MS. Although we have previously shown that a decrease in the P cycle is involved in the resistance of zhongshengmycin, another aminoglycoside antibiotic, and the exogenous alanine also showed a reversal effect on the antibiotic resistance in Xoo (Guan et al., 2022), the current study further reveals the dominant metabolic flux distribution of the alanine-pyruvate-P cycle *in vivo*, and also demonstrates the influence of pyruvate on KA resistance in Xoo. Our previous work and the present study expand the knowledge of the aminoglycoside antibiotic resistance mechanisms in the Xoo, and may be helpful in providing a new approach for controlling aminoglycoside antibiotic-resistant Xoo from a metabolic perspective.

## Materials and methods

### Bacterial strains and cultivation

The wild-type strain of *Xanthomonas oryzae* (Z173-S) in this study was obtained from the collection of our lab store. Z173-R_KA_ was one of the Z173-S-derived strains, which was selected by sequential propagations in PSA media under the stress of kasugamycin (KA) with the concentration of half of the MIC at 30°C for 13 generations. All bacterial cultures were grown in PSA solid media or broth (peptone 10 g/L, sucrose 10 g/L, sodium glutamate 1 g/L, agar 2% for solid media, pH 7.0).

### Determination of the MIC of Z173-S and Z173-R_KA_

The minimum inhibitory concentration (MIC) measurement was as previously described (Wang et al., 2021). In brief, a single colony of Z173-S or Z173-R_KA_ was picked to 3 mL PSA broth and incubated overnight at 30°C, and then the 1% (v / v) seed solution was inoculated in 24-deep-well plates equipped with 2 mL PSA broth media and KA for 18 h, where the concentration gradient of KA was set to 0, 30, 60, 120, 240, 480, 960, and 1,920 μg/mL, and cultured at 600 rpm, 30°C. The OD_600_ value was measured by the Multiskan Sky microplate reader. Each concentration contained at least three biological replicates.

### Sample preparation

After the overnight culture of *Xanthomonas oryzae*, the single colonies were cultured in the liquid media containing 50 mL PSA to OD_600_ = 1.0. After 4°C, 10,000 rpm centrifuge 5 min, the appropriate amount of Z173-S and Z173-R_KA_ cells were collected. The supernatant was discarded, and the precipitate was washed with 0.9% saline three times. The metabolic process of the cells was then stopped by adding 10 mL of saline and a 2-fold volume of chromatographic grade methanol (pre-cooled at −20°C for more than 24 h) and left for at least 1 h. The supernatant was discarded by centrifugation, and the bacterial cells were collected and frozen at −80°C. Each sample contained five biological repeats.

### GC-MS analysis

The samples were resuscitated with 500 μL frozen methanol, and 10 μL 0.2 mg/mL ribitol alcohol was added as the internal standard. After breaking 5 min under 120 W ultrasound, the cells were centrifuged for 10 min at 4°C and 12,000 g. 500 μL supernatant was dried with a nitrogen-blowing apparatus, followed by carbonyl functions protection with 80 μL (20 mg/mL) methoxyamine hydrochloride in pyridine through a 30 min 37°C reaction. After that, the sample was derivatized with 80 μL N-methyl-N-trimethylsilyltrifluoroacetamide (MSTFA, Sigma-Aldrich) in 200 rpm incubation at 37°C for 30 min. Finally, the sample was centrifuged at 12,000 g for 5 min at 4°C. 120 μL supernatant was used on the machine.

### Data processing

Chemical analysis of the derivatized samples was performed by Agilent G1701EA GC–MSD ChemStation (Agilent). The injection port temperature was maintained at 270°C, and 1 μL aliquot was injected into a dodecyl benzene sulfonic acid (DBS) column (30 m length × 250 μm i.d. × 0.25 μm thickness) using the splitless model. The MS maintains the source temperature at 250°C in the EI (direct ionization) mode at 70 eV ionization energy and 8,000 V acceleration voltage. The MS quadrupole temperature remains constant at 150°C. The initial temperature of the GC oven was set to 85°C for 3 min, followed by an increase to 285°C at a rate of 5°C/min. After that, the temperature was increased to 310°C at a rate of 20°C/min and held for 7 min. Helium gas is used as a carrier gas. The flow remains constant at 1 ml per min, and MS was operated in the range of 50–600 m/z.

### Bioinformatics analyses

Statistical analysis was performed as described previously (Guan et al., 2021). The relative abundance of metabolites is scaled according to the total abundance of all metabolites in the sample. Compounds were identified by matching them with data from the National Institute of Standards and Technology (NIST) library and NIST MS search 2.0 program, and the mass fragmentation spectrom data was analyzed by X Calibur software (ThermoFisher, version 2.1, Waltham, MA, United States). The data were normalized by the total amount of correction and standardized data including metabolites, retention time, and peak area. IBM SPSS statistics 19 software was used to analyze significant differences in the standardized data and screen differential metabolites with *p* < 0.05. Hierarchical clustering was completed in the R platform with the package gplots using the distance matrix. The Z-score was obtained by calculating the mean and standard deviation of the experimental data. The metabolic pathways were enriched by MetaboAnalyst4.0. GraphpadPrism7 software was used for the editing and layout of the graphics.

### Antibiotic bactericidal experiments

The antibiotic bactericidal experiments were performed as previously described (Guan et al., 2022). In short, a single bacterial colony of Xoo was propagated in 50 mL of PSA broth overnight at 30°C in a shaker. The culture was centrifuged at 8,000 rpm for 5 min and washed 3 times with 30 mL of Saline (0.9%), followed by resuspended in M9 minimal media (10.0 mM sodium acetate, 2.0 mM magnesium sulfate, 0.1 mM calcium chloride), adjusting OD_600_ to 0.2. M9 media was used to test the effects of different metabolites on Xoo. To determine the effect of exogenous addition on KA-killing efficiency, we applied alanine (40 mM)/furfural (0.17 μL/mL)/sodium pyruvate (60 mM)/D-alanine (60 mM) to the medium with KA, and the Xoo was cultured at 30°C, 200 rpm for 6 h. After incubation, 100 μL of samples were transferred and serially diluted. An aliquot of 5 μL of each dilution was plated in PSA agar media and incubated at 30°C for 36–48 h. The percent survival is determined by dividing the CFU obtained from the treated sample by the CFU obtained from the control. Only dilutions that yielded 20 to 200 colonies were available for calculation.

### Detection of the P cycle-associated enzyme activities and pyruvate content

It has been reported that SDH, α-KGDH and PDH are three key enzymes of the P cycle (Su et al., 2018, 2020). We used SDH, α-KGDH, and PDH detection kits (Solabio, Beijing, China) to measure the efficiency of the P cycle in Xoo. The strains were cultured with or without alanine/furfural/sodium pyruvate for 24 h, followed by centrifugation at 4°C, 8,000 rpm for 5 min. The bacterial cells were disrupted by an ultrasonic crusher at 60% strength. After that, the supernatant was obtained by centrifugation for 10 min at 4°C and 11,000 g. The protein concentration was used to calculate the enzyme activity, which was determined by BCA Protein Assay Kit (Dalian meilunbio biotechnology company, Dalian, China).

The pyruvate content was determined by the pyruvate content detection kit (Solabio, Beijing, China). Briefly, the Z173-R_KA_ sample was cultured, collected, and disrupted according to the same method described above in the enzyme activity assay. After that, the supernatant was used for pyruvate content measuring following the specification.

### Quantitative real-time PCR

The total RNA of *Xanthomonas oryzae* was extracted according to the Biospin Total RNA Extraction Kit specification. Firstly, the single colony was inoculated in the test tube with 3 ml seed medium and cultured in a shaker at 30°C, 200 rpm to OD_600_ = 0.6. 2 mL bacterial solution was transferred to a 2 mL enzyme-free centrifuge tube. Secondly, the concentration and purity of total RNA extracted by the above method were determined by full-wavelength enzyme labeling instrument μDrop plate (Thermo, United States), and then the cDNA was synthesized and reverse transcribed (TransGen Biotech, Beijing, China). Reverse transcription was carried out on a 96-well plate with 20 μL per sample well. The primers used for qRT-PCR are shown in Supplementary Table S1. At least three biological replicates were carried out for each experimental sample.

## Results

### Screening of the drug-resistant strain

**Figure 1:**
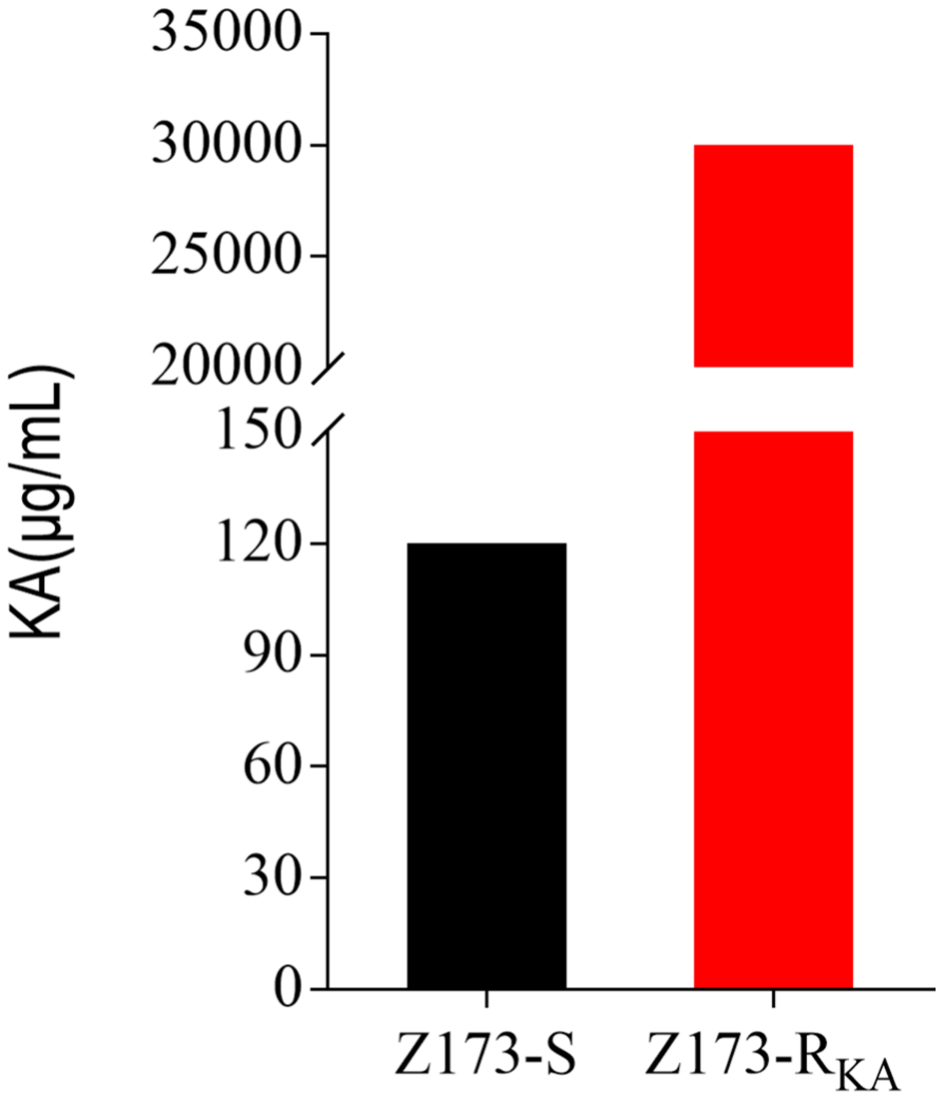
MIC of KA in Z173-S and Z173-R_KA_. Z173-S and Z173-R_KA_ were incubated with PSA broth containing different concentrations of KA in 24-well plates at 30°C for 18 h. Results are displayed as mean ± SD. At least three biological repeats were carried out.

The minimum inhibitory concentration (MIC) of wild-type *Xanthomonas oryzae* (Z173-S, preserved in the laboratory) against Kasugamycin (KA) was 120 μg/mL. A single colony of Z173-S was inoculated on PSA solid medium containing KA for continuous passage. After 13 passages, a KA-resistant strain (Z173-R_KA_) with the MIC to KA of 30,000 μg/mL was screened out, which had a 250-fold increased resistance to KA when compared to the parent strain (Figure 1). In the following experiments, Z173-S and Z173-R_KA_ were used to study the resistance mechanism of *Xanthomonas oryzae* to KA.

### Metabolic profiles of Z173-S and Z173-R_KA_

**Figure 2:**
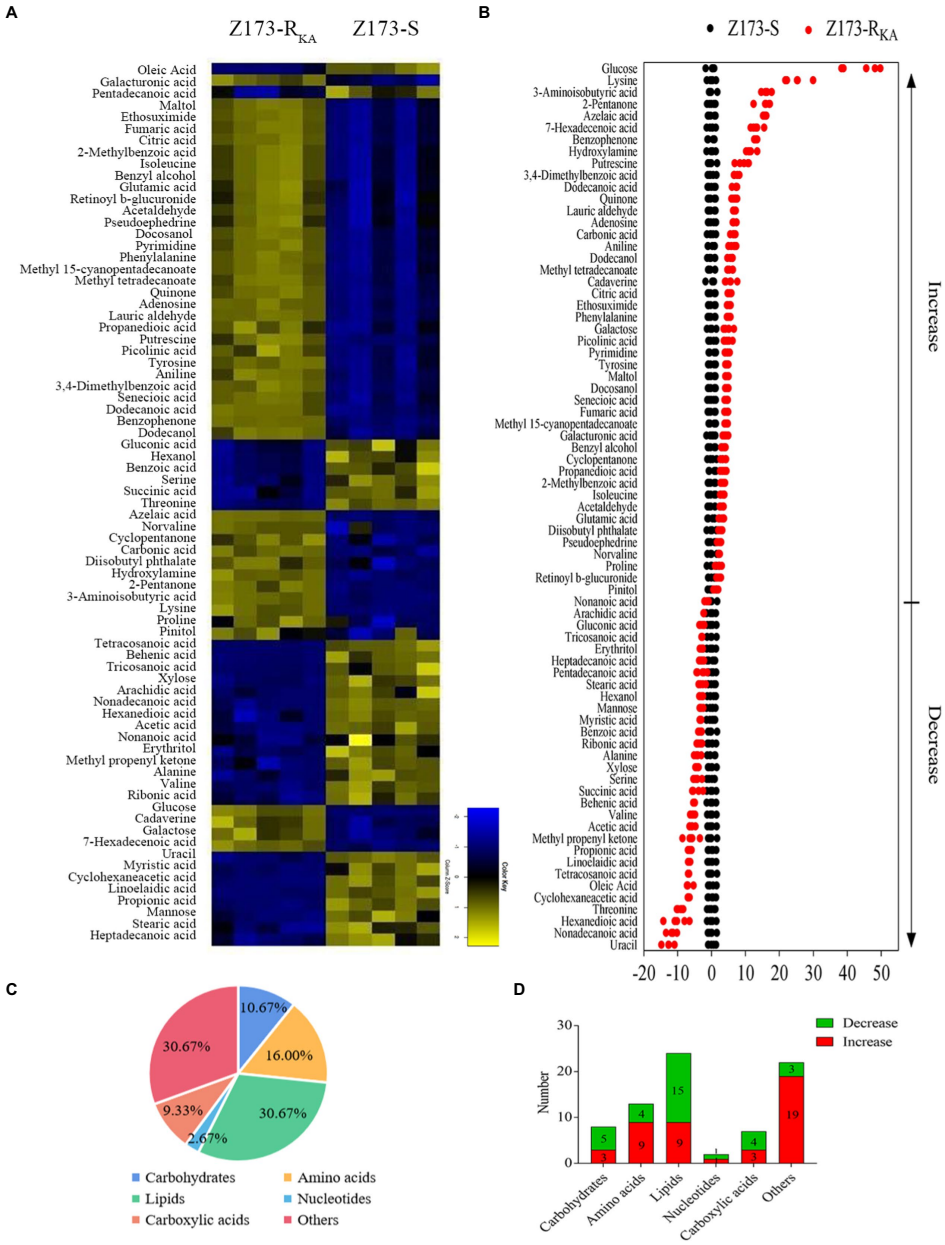
Identification of differential metabolites in Z173-S and Z173-R_KA_. **(A)** Heat map of unsupervised hierarchical clustering of varied abundance of metabolites. Yellow and blue indicate increase and decrease of metabolites relative to the median metabolite level, respectively (see color scale). **(B)** Z-score plots. Each point represents one metabolite in one technique repeat. Black and red represent Z173-S and Z173-R_KA, respectively_. **(C)** Category of varied abundance of metabolites. **(D)** Number of metabolites increased and decreased in six categories.

To unveil the metabolic difference between Z173-S and Z173-R_KA_, GC–MS-based metabolomics was used to obtain the global metabolites in these two strains. A total of 92 metabolites were identified, and we clustered biological replicates of all metabolites in this sample group to obtain a global metabolite clustering heatmap (Supplementary Figure S1A). These identified metabolites can be categorized into carbohydrates (10.87%), amino acids (15.22%), lipids (33.70%), nucleotides (2.17%), carboxylic acids (8.70%) and others (29.35%; Supplementary Figure S1B). Seventy-five differential metabolites were detected between Z173-S and Z173-R_KA_ based on the *p* < 0.05, and the clustering heatmap of these differential metabolites was shown in Figure 2A. Among the 75 deferential metabolites, 45 were increased, and 30 were decreased in Z173-R_KA_ compared to that in Z173-S (Figure 2B). These differential metabolites can be categorized into carbohydrates (10.67%), amino acids (16.00%), lipids (30.67%), nucleotides (2.67%), carboxylic acid (9.33%) and others (30.67%), respectively (Figure 2C). The numbers of upregulated or downregulated differential metabolite numbers in each category in Z173-R_KA_ were shown in Figure 2D. These results suggest that the metabolism of Z173-R_KA_ undergoes substantial changes, which may be related to the high resistance of Z173-R_KA_ to KA.

### Differential metabolic pathway analysis

**Figure 3:**
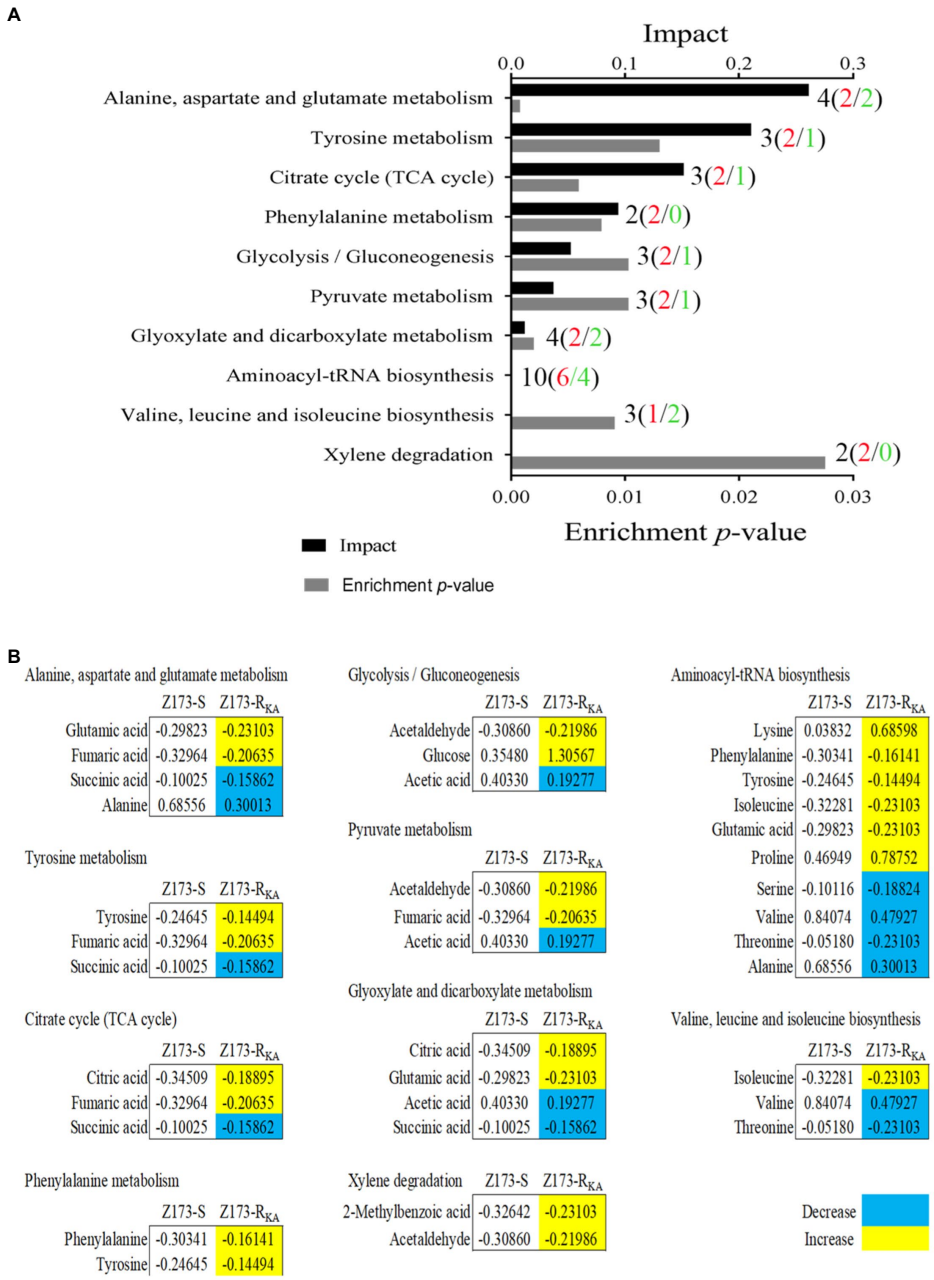
Pathway enrichment analysis. **(A)** Pathways are enriched in differential abundance of metabolites between Z173-S and Z173-R_KA_. Significantly enriched pathways are selected to plot (*p*-value < 0.05), and their impact was indicated. Black, red, and green fonts of the labels refer to the number of the total, upregulated, and downregulated differential metabolites in each metabolic pathway, respectively. **(B)** The relative abundance of metabolites of each pathway listed in **(A)**. Numbers show the relative values of differential metabolites. Yellow color and blue color indicate increased and decreased metabolites, respectively.

To better understand the metabolic difference between Z173-S and Z173-R_KA_, MetaboAnalyst^1^ was used to conduct pathway enrichment analysis on the differential metabolites. With *p* < 0.05 as the significance criterion, 10 differential metabolic pathways were significantly enriched, including alanine, aspartate and glutamate metabolism; citrate cycle (TCA cycle); glyoxylate and dicarboxylate metabolism; phenylalanine metabolism; pyruvate metabolism; glycolysis/gluconeogenesis; aminoacyl-tRNA biosynthesis; valine, leucine and isoleucine biosynthesis, and xylene degradation. The numbers of total (up/down) metabolites related to each pathway were labeled in Figure 3A. The integrative analysis of metabolites in each pathway were shown in Figure 3B. Theoretically, the upregulated or downregulated metabolites indicate an increase or decrease of the specific metabolic pathways *in vivo*, respectively. However, further experimental verification is needed to reveal the differential metabolic characterization between Z173-S and Z173-R_KA_. Noteworthily, among all the enriched pathways, the alanine, aspartate and glutamate metabolism, the tyrosine metabolism, and the TCA cycle are the top three impacted pathways involved in the Z173-R_KA_. Moreover, the TCA cycle can be promoted by the other two metabolic pathways (Zhang et al., 2019). Therefore, the TCA cycle is identified as a crucial metabolic pathway involved in the KA-resistant in Xoo.

### Depression of the P cycle in Z173-R_KA_

**Figure 4:**
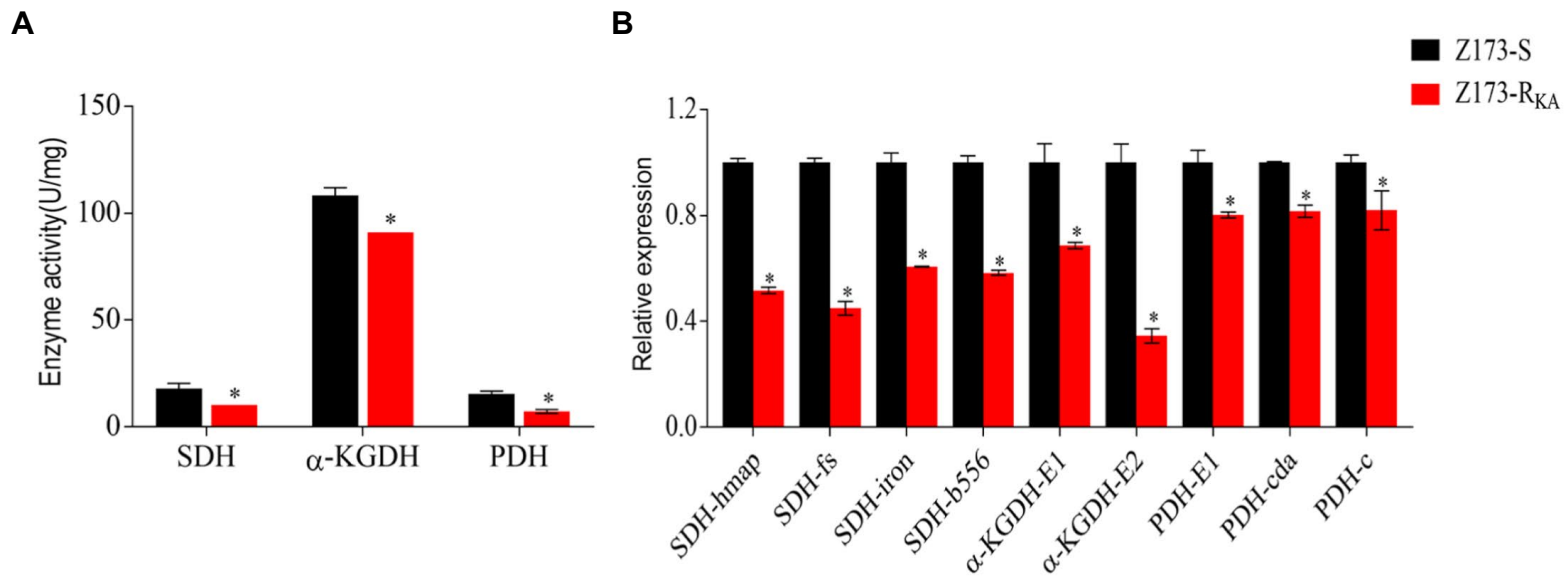
The depression of P cycle. **(A)** Enzymatic activities of SDH, α-KGDH and PDH of Z173-S and Z173-R_KA_. **(B)** Quantitative real-time PCR for gene expression of the P cycle. Results are reported as mean ± SD, and *p*-values are identified (^*^p < 0.05) as determined by the two-tailed Student’s *t*-test. At least three biological repeats were carried out.

Recently, Su et al. already showed that the TCA cycle is a part of the P cycle, which is associated with antibiotic resistance developing in various bacteria (Li et al., 2019; Su et al., 2021). To explore the relationship between the P cycle and KA-resistance in Z173-R_KA_, the activities of three key enzymes of the P cycle, i.e., succinate dehydrogenase (SDH), α-ketoglutarate dehydrogenase (α-KGDH) and pyruvate dehydrogenase (PDH) were measured. Interestingly, the activities of SDH, α-KGDH, and PDH were significantly lower in Z173-R_KA_ compared to that in Z173-S, indicating a depressed P cycle in Z173-R_KA_ (Figure 4A). Furthermore, the transcriptional levels of the genes encoding these three enzymes showed that nine genes related to SDH, α-KGDH, and PDH were all significantly downregulated, which was consistent with enzyme activity (Figure 4B). These results suggested that the downregulation of the P cycle was the metabolic characteristic in the KA-resistant Xoo, Z173-R_KA_.

### Inhibition of the P cycle elevates the resistance of Xoo to KA

**Figure 5:**
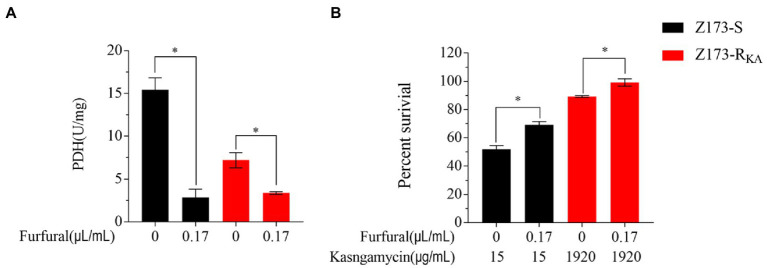
Analysis of P cycle in the presence of inhibitor. **(A)** Enzymatic activity of PDH of Z173-S and Z173-R_KA_ in the presence or absence of 0.17 μL/mL furfural. **(B)** Percentage of survival of Z173-S and Z173-R_KA_ in the presence of the indicated concentrations of furfural plus 15/1,920 μg/mL KA. Results are reported as mean ± SD, and *p*-values are identified (^*^p < 0.05) as determined by the two-tailed Student’s *t*-test. At least three biological repeats were carried out.

As the depression of the P cycle was the characteristic of Z173-R_KA_, we hypothesized that inhibition of the P cycle would elevate the KA-resistant in Xoo. Therefore, furfural, a noncompetitive inhibitor of PDH, was used to repress the intracellular P cycle (Su et al., 2018; Guan et al., 2021). With the presence of furfural, the activities of PDH in Z173-S and Z173-R_KA_ were both significantly reduced (Figure 5A), indicating a furfural-dependent P cycle depression. In addition, the bacterial survival percentage of Z173-S and Z173-R_KA_ with or without the inhibitor were assayed. As a result, the survival percentage of Z173-S and Z173-R_KA_ with the furfural addition were all higher than that without the furfural (Figure 5B). These findings confirmed that the depressed P cycle was associated with the KA resistance in Xoo.

### Exogenous alanine increases pyruvate content and enhances P cycle-associated KA-mediated killing

**Figure 6:**
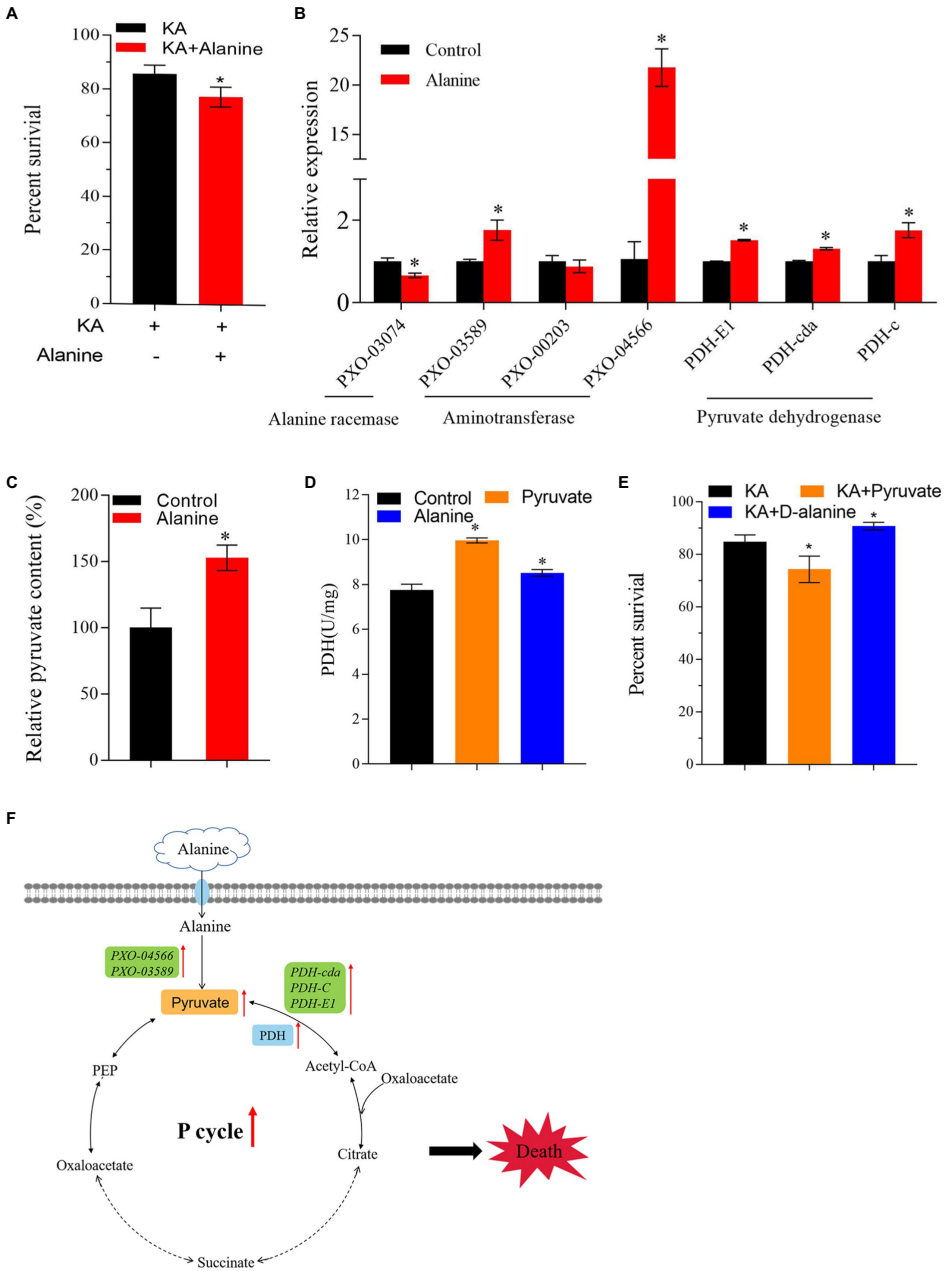
Exogenous Alanine reverses the KA resistance of Xoo-R_KA._ **(A)** Percentage of survival of Z173-R_KA_ in the presence or absence of 40 mmol/L alanine plus 960 μg/mL KA. **(B)** The transcriptional levels of alanine racemase, aminotransferase and PDH of Z173-R_KA_ in the presence or absence of 40 mmol/L alanine. **(C)** The relative pyruvate content of Z173-R_KA_ in the presence or absence of 40 mmol/L alanine. **(D)** Enzymatic activity of PDH of Z173-R_KA_ in the presence or absence of 60 mmol/L pyruvate/40 mmol/L alanine. **(E)** Percentage of survival of Z173-R_KA_ in the presence or absence of 60 mmol/L pyruvate/60 mmol/L/D-alanine plus 960 μg/mL KA. **(F)** Metabolic pathway of alanine into the P cycle. Orange, blue and green shades represent content, enzyme activity and gene transcriptional levels, respectively. Results are reported as mean ± SD, and *p* values are identified (^*^p < 0.05) as determined by the two-tailed Student’s *t*-test. At least three biological repeats were carried out.

Previous studies showed that the intracellular metabolomic status could be changed by exogenous additions, turning the antibiotic-resistant bacterium into an antibiotic-sensitive bacterium (Su et al., 2015; Rouillard et al., 2021; Yong et al., 2021). In the present study, alanine, aspartate and glutamate metabolism is the most affected differential metabolic pathway. Moreover, the metabolic abundance of alanine is significantly downregulated. Therefore, alanine might be a crucial metabolite that affects the KA-mediated killing efficiency in Xoo. In addition, alanine metabolism was linked with the production of pyruvate and acetyl-CoA, which flow to the P cycle *in vivo*. Therefore, we speculated that exogenous alanine could affect the metabolic activity of Z173-R_KA_ and enhance the killing efficiency of KA on Z173-R_KA_. Our work confirmed this hypothesis by adding exogenous alanine in the bactericide experiment. The result showed that the KA-mediated killing efficiency was significantly enhanced with the exogenous alanine, indicating that alanine could reverse the KA resistance in Z173-R_KA_ (Figure 6A).

As known to us, alanine may transform into pyruvate or D-alanine under the action of aminotransferase or alanine racemase, respectively. To figure out the dominant converting direction of alanine *in vivo*, the transcriptional levels of genes encoding aminotransferase and alanine racemase were measured. The result showed that the alanine racemase gene transcription level was downregulated, and the transcription levels of genes encoding aminotransferase and PDH were significantly upregulated (Figure 6B). In addition, the increased pyruvate content was detected after alanine addition (Figure 6C). The activity of PDH was also raised by exogenous pyruvate or alanine (Figure 6D). These results indicate that alanine flows predominantly to pyruvate rather than to D-alanine *in vivo*.

On the other hand, the effect of pyruvate or D-alanine on the KA-killing efficiency to Z173-R_KA_ was also assayed. Interestingly, pyruvate improves the KA-killing efficiency, while the D-alanine improves the survival percentage of Z173-R_KA_ under KA stress (Figure 6E). Overall, our work demonstrates that the alanine is likely to enhance the killing efficiency of KA through the alanine-pyruvate-P cycle pathway rather than through D-alanine in Z173-R_KA_ (Figure 6F).

## Discussion

The GC-MS-based metabolomic analysis has been proven to be a practical approach to investigating bacterial antibiotic-resistance mechanisms. It feedbacks the changes in the type, quantity, and regularity of all low-molecular-weight metabolites (including amino acids, sugars and phosphate sugars, biogenic amines, and lipids) of drug-resistant strains during a specific physiological period (Ye et al., 2018; Hani et al., 2021). Various studies have shown that the metabolic spectrum of many drug-resistant bacteria fluctuates when compared to the drug-sensitive ones, affecting the sensitivity of drug-resistant bacteria to drugs (Su et al., 2018; Li et al., 2020). In the present study, we adopted the GC–MS-based metabolomics to analyze the metabolomes of wild-type *Xanthomonas oryzae* (Z173-S) and Kasugamycin (KA)-resistant strain (Z173-R_KA_) and identify significant affected metabolic pathways which may be involved in KA resistance in Z173-R_KA_. We found that the metabolic spectrums in the KA-resistant and KA-sensitive strains are very different, and the low flux P cycle may result in the KA resistance in Z173-R_KA_. In addition, we also proved that alanine is a key differential metabolite, which can restore the sensitivity of Z173-R_KA_ to KA and further promote the KA-mediated killing efficiency by improving the P cycle.

Firstly, we noticed that the metabolism of KA-resistant strain Z173-R_KA_ undergoes substantial changes. Taking Z173-S as the control group and Z173-R_KA_ as the experimental group, 75 differential metabolites were identified, and 10 significantly differential metabolic pathways were enriched. Of particular interest is that the three most affected metabolic pathways are all related to the TCA cycle, which is a part of the P cycle. This finding is consistent with the recent reports that the inhibition of the P cycle affects the uptake of antibiotics. For example, the reduction of respiration efficiency, P cycle and membrane proton motive force, and the increase of fatty acid biosynthesis mediated the resistance of *Vibrio alginolyticus* to ceftazidime (Liu et al., 2019). The depression of the P cycle led to the resistance of *Vibrio alginolyticus* to gentamicin (Zhang et al., 2019). The inactivation of the P cycle and the fatty acid biosynthesis promoted the resistance of *Vibrio alginolyticus* to ofloxacin (Yin et al., 2022).

Secondly, two aspects of experiments were conducted to verify that the depressed P cycle was associated with the KA resistance in Xoo. Firstly, the activities of three key enzymes in the P cycle and the transcriptional levels of their encoding genes were measured. The activities of three key enzymes, i.e., SDH, α-KGDH, and PDH of the P cycle, were notably reduced in Z173-R_KA_, which were confirmed by the downregulation of related genes transcriptional levels, indicating that the suppressed P cycle contributed to the resistance of Z173-R_KA_ to KA. Secondly, the effect of exogenous inhibitors on the P cycle and antibiotic-killing efficiency was assayed. With the supplementary furfural (an inhibitor of PDH on the P cycle) in the culture medium, the activities of PDH in Z173-S and Z173-R_KA_ were downregulated, and the killing effect mediated by KA was weakened. These results support the conclusion that the depression of the P cycle leads to the resistance of Xoo to KA. To our knowledge, there are rare reports on the mechanism of KA resistance in *Xanthomonas oryzae,* and our work seems to be the first exploration to study the mechanism of KA resistance in *Xanthomonas oryzae* by metabonomics based on GC–MS.

Recently, numerous studies have shown that exogenous metabolites or crucial biomarkers can enhance the killing effect of antibiotics on drug-resistant strains by affecting metabolic pathways and energy changes (Yang et al., 2018; Kuang et al., 2021a; Chung et al., 2022). In the present study, the enrichment analysis of metabolic pathways and the change of metabolites abundance indicated that alanine might be a crucial candidate associated with the enhancement of KA-mediated killing efficiency in Z173-R_KA_. Our experimental results showed that exogenous alanine could enhance the KA-killing efficiency to Z173-R_KA_. In addition, the transcriptional level of genes encoding aminotransferase and PDH, the pyruvate content, and PDH activity were increased with the alanine addition. Besides, pyruvate could also increase PDH activity and bactericidal effect. These findings suggest that the alanine is likely to restore the sensitivity of Z173-R_KA_ to KA and reduce the survival rate of the strain through alanine-pyruvate-P cycle pathway.

Interestingly, we also found that D-alanine could improve the survival of Z173-R_KA_, this may be attributed to the promotion of peptidoglycan biosynthesis caused by exogenous D-alanine addition, thus blocking the entry of antibiotics and increasing the survival rate of drug-resistant bacteria (Peschel et al., 2000; Chacon et al., 2002). However, the antibiotic resistance in the microbes is complicated and more metabolic pathways or metabolites involved in the KA resistance in Xoo could be explored in the future. The present results are similar to the previous reports that alanine can weaken the antibiotic resistance of bacteria by affecting a specific metabolic pathway. In *Edwardsiella tarda*, alanine or glucose enhances the kanamycin-mediated killing efficiency by promoting the TCA cycle and increasing the production of NADH and proton motive force (Peng et al., 2015). Our previous study also revealed that exogenous alanine stimulates the killing efficiency of zhongshengmycin to *Xanthomonas oryzae* by activating the P cycle (Guan et al., 2022). Alanine also heightens the killing effect of gentamicin on *Vibrio alginolyticus* by reducing the level of NO (Kuang et al., 2021a). However, to our knowledge, this study is the first to demonstrate that alanine restores Xoo’s sensitivity to KA and reduces the survival rate of the strain through the alanine-pyruvate-P cycle pathway rather than other pathways. In addition, we found that the KA and other antibiotics studied in the reports mentioned above, such as kanamycin, zhongshengmycin and gentamicin, all belong to aminoglycoside antibiotics. Therefore, alanine may have a similar effect on bacterial resistance to aminoglycoside antibiotics, but the specific conclusion needs further experimental proof.

Notably, the resistant strain used in this study was obtained by laboratory screening. In recent years, artificial drug-resistant strains have been used in various bacterial antibiotic resistance mechanism studies (Jiang et al., 2020; Li et al., 2020; Tang et al., 2021). Previous studies also showed that the wild-type drug-resistant samples often showed similar metabolic characteristics to the artificial ones (Kuang et al., 2021b, 2022). On the other hand, the work on isolating the wild-type Xoo with high drug resistance (Xoo-Rwide) is necessary, and the metabolic analysis should be carried out with the Xoo-Rwide in further study to verify whether the P cycle is involved in the KA-resistance in Xoo-Rwide from the farm or not.

In conclusion, this study unveiled that the P cycle is an important element for developing the KA resistance in Xoo. By measuring the changes of key enzyme activities and related gene transcriptional levels of the P cycle, as well as the effect of the PDH inhibitor furfural on this pathway, we further verify that the inhibited P cycle is the key metabolic characteristic involved in KA resistance in Xoo. Furthermore, we confirmed that exogenous alanine, as a key differential metabolite, could enhance the KA-mediated killing effect and restore the sensitivity of Z173-R_KA_ to KA by activating the P cycle. Our work innovatively unveils the KA-resistance developing mechanism in Xoo, provides a new idea for developing metabolic regulation to address and shows great significance for solving the antibiotic resistance problem in bacterial control in the agricultural field.

## Data availability statement

The datasets presented in this study can be found in online repositories. The names of the repository/repositories and accession number(s) can be found in the article/Supplementary material.

## Author contributions

ML and YG: conceptualization, methodology, formal analysis and writing—original draft preparation. PS and ZZ: software and validation. ML, PS, and ZZ: investigation and data curation. YG: resources, writing—review and editing, supervision, project administration, and funding acquisition. ML: visualization. All authors contributed to the article and approved the submitted version.

## Funding

This research was funded by the National Natural Science Foundation of China (32072481).

## Conflict of interest

The authors declare that the research was conducted in the absence of any commercial or financial relationships that could be construed as a potential conflict of interest.

## Publisher’s note

All claims expressed in this article are solely those of the authors and do not necessarily represent those of their affiliated organizations, or those of the publisher, the editors and the reviewers. Any product that may be evaluated in this article, or claim that may be made by its manufacturer, is not guaranteed or endorsed by the publisher.

## Footnotes

^1^ https://www.metaboanalyst.ca/
