# Supplementary figures and images for: Alanine-mediated P cycle boosting enhances the killing efficiency of kasugamycin on antibiotic-resistant Xanthomonas oryzae
Source: Front Microbiol. 2023 Apr 18;14:1160702. doi: 10.3389/fmicb.2023.1160702 (PMC10151481; doi:10.3389/fmicb.2023.1160702)

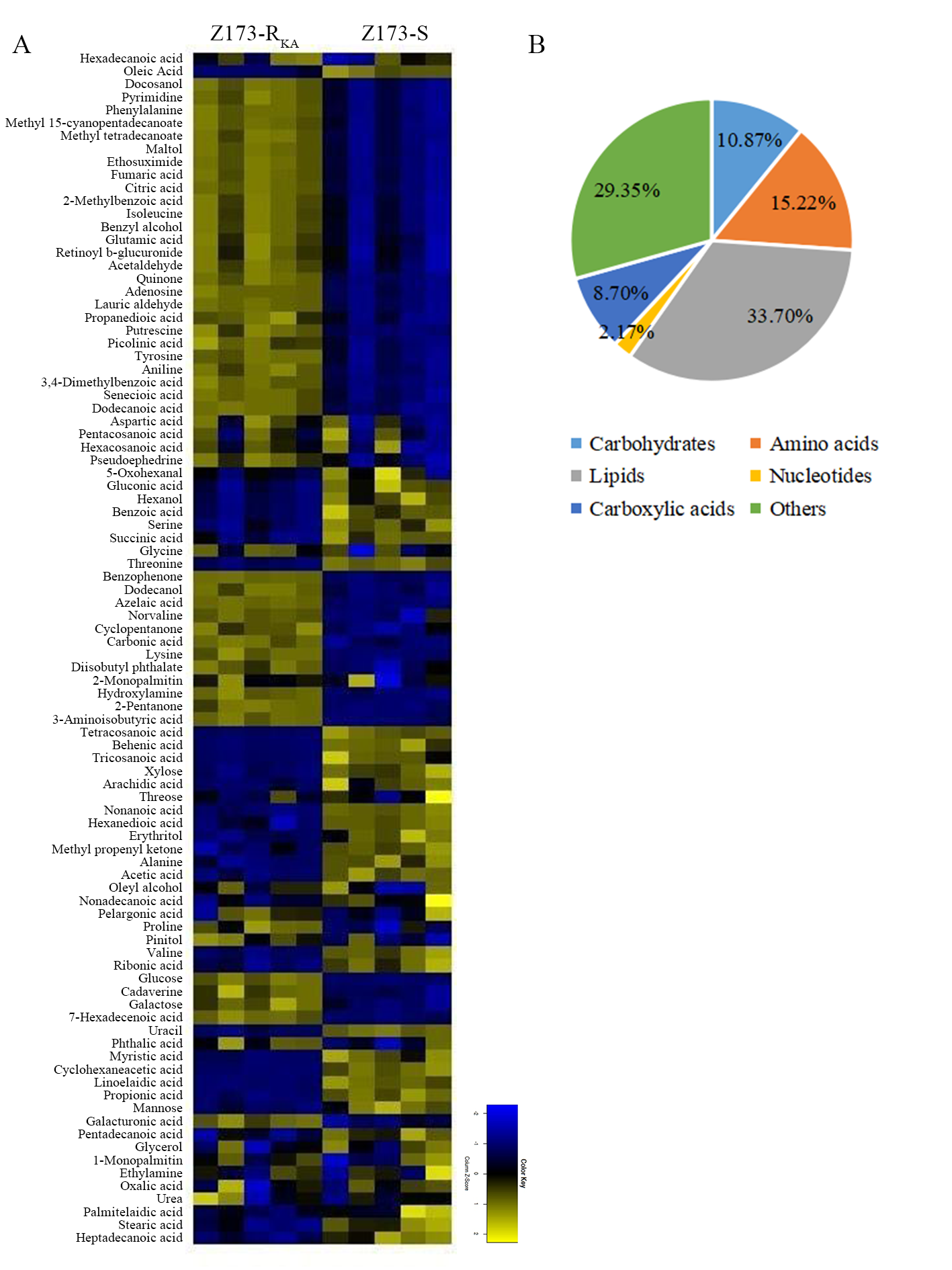

Supplement: Supplementary file 1 [file Image_1.TIF]
