# Supplementary material for: Alanine-mediated P cycle boosting enhances the killing efficiency of kasugamycin on antibiotic-resistant Xanthomonas oryzae
Source: Front Microbiol. 2023 Apr 18;14:1160702. doi: 10.3389/fmicb.2023.1160702 (PMC10151481; doi:10.3389/fmicb.2023.1160702)
Supplement: Supplementary file 2 [file Table_1.docx]

# Supplementary Material

**Table S1. The primers used in this study for qRT-PCR.**

| **Primers** | **Sequences (5’-3’)** | **Annotation** |
| --- | --- | --- |
| qRT-16s-F  qRT-16s-R | AACACTGACACTGAGGCACGAAAG  CCCAGGCGGCGAACTTAACG | 16S-*Xanthomonas oryzae* strain LMG 5047 16S ribosomal RNA |
| OGDH-E1-F  OGDH-E1-R | GCACGACCAGAAGACCGACAAC  CCATCACCGCTTCTTCGCTGAG | 2-oxoglutarate dehydrogenase, E1 component |
| OGDH-E2-F  OGDH-E2-R | GCCAGTCGTTCGCCGATGTC  AAGGTGCCGCCATTGGTGATG | 2-oxoglutarate dehydrogenase, E2 component |
| SDH-hmap-F  SDH-hmap-R | GCAGGTGGTGCTGGAAGATTACG  ACGGCAAACACGCTGACGATG | Succinate dehydrogenase, hydrophobic membrane anchor protein |
| SDH-fs-F  SDH-fs-R | GCTGCTGGATCTGGTGGTGTTC  AAGGTCTTGTGCGGCTGATTGG | Succinate dehydrogenase, flavoprotein subunit |
| SDH-b556-F  SDH-b556-R | CGTTCGTCGGCATGGGTTGG  TACGGCACCTCCAGACAGCAG | Succinate dehydrogenase, cytochrome b556 subunit |
| SDH-iron-F  SDH-iron-R | TGAACATCGACGGCACCAATACG  GCAGCGGATAGATCGGCACTTC | Succinate dehydrogenase iron-sulfur protein |
| PDH-cda-F  PDH-cda-R | AGAGCCTGGTCACCCTGGAATC  ACCACACTGCCCTGCGAGAG | Pyruvate dehydrogenase complex dihydrolipoamide acetyltransferase |
| PDH-E1-F  PDH-E1-R | CGTGCCTTTGCTGACCAGATCC  TCCGAGCGACCGAACCCATC | PDH E1component |
| PDH-c-F  PDH-c-R | CGGCAAGGAATACGTGGAGTGG  AAGGTGTCGCAGTTGAGCATCG | PDH cytochrome |
| PXO-04566-F  PXO-04566-R | GACGGCTCGATCTGGACCTTC  CCACGGCGACCAACTGACG | Aminotransferase |
| PXO-00203-F  PXO-00203-R | GGATGCGTTGGCGTTGATTCTG  CCGGCACCAGCACCAGATG | Aminotransferase |
| PXO-03589-F  PXO-03589-R | GCGGCGGCAATCGGAAATTC  TCAGCGTCGGGCACAAATGG | Aminotransferase |
| PXO-03074-F  PXO-03074-R | CAGCACCAGCGAGGCGATC  TGCGGGTGTGGTTGAAGATGG | Alanine racemase |
